# *N*-(3,4-Dimethyl­phen­yl)-4-methyl­benzene­sulfonamide

**DOI:** 10.1107/S1600536809010459

**Published:** 2009-03-28

**Authors:** B. Thimme Gowda, Sabine Foro, P. G. Nirmala, Hiromitsu Terao, Hartmut Fuess

**Affiliations:** a Department of Chemistry, Mangalore University, Mangalagangotri 574 199, Mangalore, India; b Institute of Materials Science, Darmstadt University of Technology, Petersenstrasse 23, D-64287 Darmstadt, Germany; c Faculty of Integrated Arts and Sciences, Tokushima University, Minamijosanjima-cho, Tokushima 770-8502, Japan

## Abstract

In the crystal structure of the title compound, C_15_H_17_NO_2_S, the conformations of the N—C bond in the C—SO_2_—NH—C segment are *trans* and *gauche*, respectively, with respect to the S=O bonds. The mol­ecule is bent at the S atom with a C—SO_2_—NH—C torsion angle of −61.8 (2)°. Furthermore, the conformation of the N—H bond and the 3-methyl group in the aniline benzene ring are nearly *anti* to each other. The dihedral angle between the benzene rings is 47.8 (1)°. In the crystal, N—H⋯O hydrogen bonds link the molecules into chains.

## Related literature

For the preparation of the compound, see: Shetty & Gowda (2005 ▶). For related structures, see: Gelbrich *et al.* (2007 ▶); Gowda *et al.* (2008*a* ▶,*b* ▶; 2009 ▶); Perlovich *et al.* (2006 ▶)
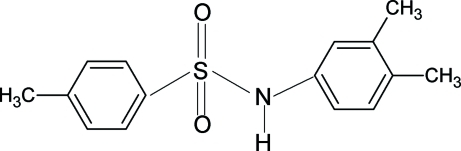


## Experimental

### 

#### Crystal data

C_15_H_17_NO_2_S
*M* *_r_* = 275.36
Monoclinic, 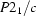

*a* = 9.2528 (7) Å
*b* = 15.329 (1) Å
*c* = 10.4469 (7) Å
β = 102.558 (7)°
*V* = 1446.30 (17) Å^3^
*Z* = 4
Mo *K*α radiation
μ = 0.22 mm^−1^
*T* = 299 K
0.45 × 0.40 × 0.34 mm

#### Data collection

Oxford Diffraction Xcalibur with Sapphire CCD detector diffractometer
Absorption correction: multi-scan (*CrysAlis RED*; Oxford Diffraction, 2007 ▶) *T*_min_ = 0.907, *T*_max_ = 0.929
10438 measured reflections
2902 independent reflections
2360 reflections with *I* > 2σ(*I*)
*R*_int_ = 0.014

#### Refinement

*R*[*F* ^2^ > 2σ(*F* ^2^)] = 0.042
*wR*(*F* ^2^) = 0.127
*S* = 1.06
2902 reflections
175 parameters
H-atom parameters constrained
Δρ_max_ = 0.49 e Å^−3^
Δρ_min_ = −0.48 e Å^−3^

### 

Data collection: *CrysAlis CCD* (Oxford Diffraction, 2004 ▶); cell refinement: *CrysAlis RED* (Oxford Diffraction, 2007 ▶); data reduction: *CrysAlis RED*; program(s) used to solve structure: *SHELXS97* (Sheldrick, 2008 ▶); program(s) used to refine structure: *SHELXL97* (Sheldrick, 2008 ▶); molecular graphics: *PLATON* (Spek, 2009 ▶); software used to prepare material for publication: *SHELXL97*.

## Supplementary Material

Crystal structure: contains datablocks I, global. DOI: 10.1107/S1600536809010459/fl2240sup1.cif

Structure factors: contains datablocks I. DOI: 10.1107/S1600536809010459/fl2240Isup2.hkl

Additional supplementary materials:  crystallographic information; 3D view; checkCIF report

## Figures and Tables

**Table 1:** Hydrogen-bond geometry (Å, °)

*D*—H⋯*A*	*D*—H	H⋯*A*	*D*⋯*A*	*D*—H⋯*A*
N1—H1*N*⋯O2^i^	0.86	2.42	2.963 (2)	122

Symmetry code: (i) 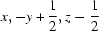.

**Fig. 1.**
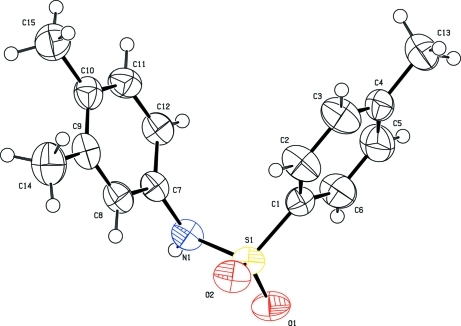
Molecular structure of the title compound, showing the atom labeling scheme. The displacement ellipsoids are drawn at the 50% probability level. The H atoms are represented as small spheres of arbitrary radii.

**Fig. 2.**
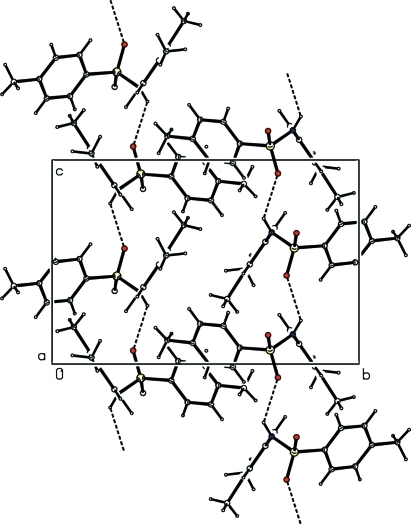
Molecular packing of the title compound with hydrogen bonding shown as dashed lines.

## supplementary crystallographic information

### Comment

As part of our study of substituent effects on the crystal structures of *N*-(aryl)-arylsulfonamides (Gowda *et al.*, 2008*a; b*; 2009), in the present work, the structure of 4-methyl-*N*-(3,4-dimethylphenyl)benzenesulfonamide (N34DMP4MBSA) has been determined. The conformations of the N—C bond in the C—SO_2_—NH—C segment of the structure are "*trans*" and "*gauche*" with respect to the S=O bonds (Fig. 1). The molecule is bent at the S atom with the C—SO_2_—NH—C torsion angle of -61.8 (2). The conformation of the N—H bond and the *meta*-methyl group in the anilino benzene ring are nearly *anti* to each other. The two benzene rings in the title compound are tilted relative to each other by 47.8 (1)°. The other bond parameters in N34DMP4MBSA are similar to those observed in *N*-(2,6-dimethylphenyl)-benzenesulfonamide (Gowda *et al.*, 2008*a*), *N*-(2,3-dimethylphenyl)- benzenesulfonamide (Gowda *et al.*, 2009), *N*-(3,5-dichlorophenyl)- benzenesulfonamide (Gowda *et al.*, 2008*b*)) and other aryl sulfonamides (Perlovich *et al.*, 2006; Gelbrich *et al.*, 2007). The N—H···O hydrogen bonds (Table 1) pack the molecules into infinite chains in the direction of *a*- axis (Fig. 2).

### Experimental

The solution of toluene (10 cc) in chloroform (40 cc) was treated dropwise with chlorosulfonic acid (25 cc) at 0 ° C. After the initial evolution of hydrogen chloride subsided, the reaction mixture was brought to room temperature and poured into crushed ice in a beaker. The chloroform layer was separated, washed with cold water and allowed to evaporate slowly. The residual 4-methylbenzenesulfonylchloride was treated with 3,4-dimethylaniline in the stoichiometric ratio and boiled for ten minutes. The reaction mixture was then cooled to room temperature and added to ice cold water (100 cc). The resultant 4-methyl-*N*-(3,4-dimethylphenyl)benzenesulfonamide was filtered under suction and washed thoroughly with cold water. It was then recrystallized to constant melting point from dilute ethanol. The purity of the compound was checked and characterized by recording its infrared and NMR spectra (Shetty & Gowda, 2005). The single crystals used in X-ray diffraction studies were grown in ethanolic solution by slow evaporation at room temperature.

### Refinement

The H atoms were positioned with idealized geometry using a riding model with C—H = 0.93–0.96 Å, N—H = 0.86 Å, and were refined with isotropic displacement parameters (set to 1.2 times of the *U*_eq_ of the parent atom). For methyl group *U*_iso_(H) = 1.5 *U*_eq_.

### Crystal data

**Table:**

C_15_H_17_NO_2_S	*F*(000) = 584
*M**_r_* = 275.36	*D*_x_ = 1.265 Mg m^−^^3^
Monoclinic, *P*2_1_/*c*	Mo *K*α radiation, λ = 0.71073 Å
Hall symbol: -P 2ybc	Cell parameters from 5065 reflections
*a* = 9.2528 (7) Å	θ = 2.3–27.3°
*b* = 15.329 (1) Å	µ = 0.22 mm^−^^1^
*c* = 10.4469 (7) Å	*T* = 299 K
β = 102.558 (7)°	Prism, colourless
*V* = 1446.30 (17) Å^3^	0.45 × 0.40 × 0.34 mm
*Z* = 4	

### Data collection

**Table:**

Oxford Diffraction Xcalibur with Sapphire CCD detector diffractometer	2902 independent reflections
Radiation source: fine-focus sealed tube	2360 reflections with *I* > 2σ(*I*)
graphite	*R*_int_ = 0.014
Rotation method data acquisition using ω and φ scans	θ_max_ = 26.4°, θ_min_ = 2.3°
Absorption correction: multi-scan (*CrysAlis RED*; Oxford Diffraction, 2007)	*h* = −11→11
*T*_min_ = 0.907, *T*_max_ = 0.929	*k* = −19→19
10438 measured reflections	*l* = −13→12

### Refinement

**Table:**

Refinement on *F*^2^	Primary atom site location: structure-invariant direct methods
Least-squares matrix: full	Secondary atom site location: difference Fourier map
*R*[*F*^2^ > 2σ(*F*^2^)] = 0.042	Hydrogen site location: inferred from neighbouring sites
*wR*(*F*^2^) = 0.127	H-atom parameters constrained
*S* = 1.06	*w* = 1/[σ^2^(*F*_o_^2^) + (0.0676*P*)^2^ + 0.5487*P*] where *P* = (*F*_o_^2^ + 2*F*_c_^2^)/3
2902 reflections	(Δ/σ)_max_ = 0.014
175 parameters	Δρ_max_ = 0.49 e Å^−^^3^
0 restraints	Δρ_min_ = −0.48 e Å^−^^3^

### Special details

**Table:**

Experimental. Empirical absorption correction using spherical harmonics, implemented in SCALE3 ABSPACK scaling algorithm.
Geometry. All e.s.d.'s (except the e.s.d. in the dihedral angle between two l.s. planes) are estimated using the full covariance matrix. The cell e.s.d.'s are taken into account individually in the estimation of e.s.d.'s in distances, angles and torsion angles; correlations between e.s.d.'s in cell parameters are only used when they are defined by crystal symmetry. An approximate (isotropic) treatment of cell e.s.d.'s is used for estimating e.s.d.'s involving l.s. planes.
Refinement. Refinement of *F*^2^ against ALL reflections. The weighted *R*-factor *wR* and goodness of fit *S* are based on *F*^2^, conventional *R*-factors *R* are based on *F*, with *F* set to zero for negative *F*^2^. The threshold expression of *F*^2^ > σ(*F*^2^) is used only for calculating *R*-factors(gt) *etc*. and is not relevant to the choice of reflections for refinement. *R*-factors based on *F*^2^ are statistically about twice as large as those based on *F*, and *R*- factors based on ALL data will be even larger.

### Fractional atomic coordinates and isotropic or equivalent isotropic displacement parameters (Å^2^)

**Table:**

	*x*	*y*	*z*	*U*_iso_*/*U*_eq_	
C1	0.0832 (2)	0.11183 (12)	0.41984 (18)	0.0420 (4)	
C2	0.2056 (3)	0.08904 (16)	0.5149 (2)	0.0618 (6)	
H2	0.2382	0.1249	0.5872	0.074*	
C3	0.2793 (3)	0.01242 (17)	0.5015 (2)	0.0676 (7)	
H3	0.3623	−0.0027	0.5653	0.081*	
C4	0.2330 (3)	−0.04188 (14)	0.3963 (2)	0.0539 (5)	
C5	0.1119 (3)	−0.01764 (17)	0.3024 (3)	0.0703 (7)	
H5	0.0800	−0.0534	0.2299	0.084*	
C6	0.0366 (3)	0.05845 (16)	0.3129 (2)	0.0640 (6)	
H6	−0.0454	0.0737	0.2481	0.077*	
C7	0.2234 (2)	0.30486 (12)	0.40062 (17)	0.0395 (4)	
C8	0.2693 (2)	0.35604 (12)	0.51153 (18)	0.0457 (5)	
H8	0.1995	0.3769	0.5560	0.055*	
C9	0.4176 (2)	0.37672 (12)	0.55727 (19)	0.0485 (5)	
C10	0.5221 (2)	0.34840 (13)	0.4878 (2)	0.0489 (5)	
C11	0.4742 (2)	0.29740 (14)	0.3767 (2)	0.0522 (5)	
H11	0.5430	0.2777	0.3304	0.063*	
C12	0.3270 (2)	0.27524 (13)	0.33332 (19)	0.0468 (5)	
H12	0.2976	0.2406	0.2592	0.056*	
C13	0.3163 (3)	−0.12473 (17)	0.3828 (3)	0.0770 (8)	
H13A	0.3446	−0.1526	0.4668	0.092*	
H13B	0.4033	−0.1111	0.3507	0.092*	
H13C	0.2540	−0.1632	0.3223	0.092*	
C14	0.4651 (3)	0.42967 (18)	0.6811 (2)	0.0709 (7)	
H14A	0.3803	0.4426	0.7167	0.085*	
H14B	0.5098	0.4831	0.6614	0.085*	
H14C	0.5355	0.3969	0.7440	0.085*	
C15	0.6836 (3)	0.37166 (18)	0.5317 (3)	0.0703 (7)	
H15A	0.7240	0.3435	0.6138	0.084*	
H15B	0.6935	0.4337	0.5422	0.084*	
H15C	0.7361	0.3525	0.4670	0.084*	
N1	0.06920 (18)	0.28438 (10)	0.35435 (15)	0.0435 (4)	
H1N	0.0181	0.3106	0.2867	0.052*	
O1	−0.15757 (16)	0.20372 (11)	0.35826 (15)	0.0568 (4)	
O2	0.02022 (16)	0.23588 (11)	0.56602 (12)	0.0528 (4)	
S1	−0.00854 (5)	0.21086 (3)	0.43102 (4)	0.04195 (17)	

### Atomic displacement parameters (Å^2^)

**Table:**

	*U*^11^	*U*^22^	*U*^33^	*U*^12^	*U*^13^	*U*^23^
C1	0.0434 (10)	0.0444 (10)	0.0383 (9)	−0.0001 (8)	0.0092 (8)	0.0027 (8)
C2	0.0734 (15)	0.0639 (14)	0.0413 (11)	0.0187 (12)	−0.0029 (10)	−0.0019 (10)
C3	0.0755 (16)	0.0693 (15)	0.0529 (13)	0.0232 (13)	0.0030 (12)	0.0091 (11)
C4	0.0597 (13)	0.0427 (10)	0.0653 (13)	0.0000 (9)	0.0264 (11)	0.0074 (10)
C5	0.0685 (15)	0.0616 (14)	0.0763 (17)	−0.0044 (12)	0.0057 (13)	−0.0236 (12)
C6	0.0542 (13)	0.0652 (14)	0.0641 (14)	0.0050 (11)	−0.0057 (11)	−0.0163 (11)
C7	0.0498 (11)	0.0377 (9)	0.0295 (8)	0.0055 (8)	0.0058 (7)	0.0037 (7)
C8	0.0596 (12)	0.0420 (10)	0.0360 (9)	0.0058 (9)	0.0114 (8)	−0.0013 (8)
C9	0.0655 (13)	0.0388 (10)	0.0375 (10)	−0.0009 (9)	0.0030 (9)	0.0002 (8)
C10	0.0520 (11)	0.0421 (10)	0.0486 (11)	0.0011 (9)	0.0022 (9)	0.0074 (9)
C11	0.0552 (12)	0.0548 (12)	0.0492 (12)	0.0074 (10)	0.0167 (10)	0.0008 (9)
C12	0.0583 (12)	0.0490 (11)	0.0326 (9)	0.0028 (9)	0.0085 (8)	−0.0041 (8)
C13	0.0853 (18)	0.0517 (13)	0.101 (2)	0.0086 (13)	0.0348 (16)	0.0037 (13)
C14	0.0883 (18)	0.0665 (15)	0.0534 (13)	−0.0132 (13)	0.0054 (12)	−0.0177 (11)
C15	0.0580 (14)	0.0666 (15)	0.0796 (17)	−0.0008 (12)	0.0001 (12)	0.0024 (13)
N1	0.0493 (9)	0.0485 (9)	0.0294 (7)	0.0078 (7)	0.0015 (7)	0.0064 (6)
O1	0.0413 (8)	0.0772 (11)	0.0495 (8)	0.0071 (7)	0.0042 (6)	−0.0027 (7)
O2	0.0600 (9)	0.0693 (9)	0.0299 (7)	0.0067 (7)	0.0118 (6)	−0.0016 (6)
S1	0.0414 (3)	0.0537 (3)	0.0298 (3)	0.0059 (2)	0.00563 (18)	−0.00009 (18)

### Geometric parameters (Å, °)

**Table:**

C1—C6	1.376 (3)	C10—C11	1.389 (3)
C1—C2	1.379 (3)	C10—C15	1.507 (3)
C1—S1	1.7557 (19)	C11—C12	1.381 (3)
C2—C3	1.380 (3)	C11—H11	0.9300
C2—H2	0.9300	C12—H12	0.9300
C3—C4	1.371 (3)	C13—H13A	0.9600
C3—H3	0.9300	C13—H13B	0.9600
C4—C5	1.370 (3)	C13—H13C	0.9600
C4—C13	1.508 (3)	C14—H14A	0.9600
C5—C6	1.375 (3)	C14—H14B	0.9600
C5—H5	0.9300	C14—H14C	0.9600
C6—H6	0.9300	C15—H15A	0.9600
C7—C12	1.382 (3)	C15—H15B	0.9600
C7—C8	1.387 (3)	C15—H15C	0.9600
C7—N1	1.438 (2)	N1—S1	1.6369 (17)
C8—C9	1.388 (3)	N1—H1N	0.8600
C8—H8	0.9300	O1—S1	1.4267 (15)
C9—C10	1.398 (3)	O2—S1	1.4296 (13)
C9—C14	1.509 (3)		
			
C6—C1—C2	119.82 (19)	C10—C11—H11	119.2
C6—C1—S1	119.82 (16)	C11—C12—C7	119.70 (18)
C2—C1—S1	120.30 (15)	C11—C12—H12	120.1
C1—C2—C3	119.3 (2)	C7—C12—H12	120.1
C1—C2—H2	120.4	C4—C13—H13A	109.5
C3—C2—H2	120.4	C4—C13—H13B	109.5
C4—C3—C2	121.5 (2)	H13A—C13—H13B	109.5
C4—C3—H3	119.3	C4—C13—H13C	109.5
C2—C3—H3	119.3	H13A—C13—H13C	109.5
C5—C4—C3	118.3 (2)	H13B—C13—H13C	109.5
C5—C4—C13	121.1 (2)	C9—C14—H14A	109.5
C3—C4—C13	120.6 (2)	C9—C14—H14B	109.5
C4—C5—C6	121.5 (2)	H14A—C14—H14B	109.5
C4—C5—H5	119.3	C9—C14—H14C	109.5
C6—C5—H5	119.3	H14A—C14—H14C	109.5
C5—C6—C1	119.6 (2)	H14B—C14—H14C	109.5
C5—C6—H6	120.2	C10—C15—H15A	109.5
C1—C6—H6	120.2	C10—C15—H15B	109.5
C12—C7—C8	119.37 (19)	H15A—C15—H15B	109.5
C12—C7—N1	120.27 (17)	C10—C15—H15C	109.5
C8—C7—N1	120.34 (17)	H15A—C15—H15C	109.5
C7—C8—C9	121.13 (19)	H15B—C15—H15C	109.5
C7—C8—H8	119.4	C7—N1—S1	119.68 (12)
C9—C8—H8	119.4	C7—N1—H1N	120.2
C8—C9—C10	119.58 (18)	S1—N1—H1N	120.2
C8—C9—C14	120.0 (2)	O1—S1—O2	119.82 (9)
C10—C9—C14	120.4 (2)	O1—S1—N1	105.59 (9)
C11—C10—C9	118.52 (19)	O2—S1—N1	106.90 (9)
C11—C10—C15	120.1 (2)	O1—S1—C1	108.88 (9)
C9—C10—C15	121.3 (2)	O2—S1—C1	107.97 (9)
C12—C11—C10	121.7 (2)	N1—S1—C1	107.01 (9)
C12—C11—H11	119.2		
			
C6—C1—C2—C3	−0.4 (4)	C14—C9—C10—C15	−1.7 (3)
S1—C1—C2—C3	−177.58 (19)	C9—C10—C11—C12	0.7 (3)
C1—C2—C3—C4	−0.4 (4)	C15—C10—C11—C12	−179.7 (2)
C2—C3—C4—C5	1.0 (4)	C10—C11—C12—C7	0.6 (3)
C2—C3—C4—C13	179.2 (2)	C8—C7—C12—C11	−0.4 (3)
C3—C4—C5—C6	−0.8 (4)	N1—C7—C12—C11	178.19 (17)
C13—C4—C5—C6	−179.0 (2)	C12—C7—N1—S1	105.46 (18)
C4—C5—C6—C1	0.0 (4)	C8—C7—N1—S1	−75.9 (2)
C2—C1—C6—C5	0.6 (4)	C7—N1—S1—O1	−177.68 (14)
S1—C1—C6—C5	177.8 (2)	C7—N1—S1—O2	53.68 (16)
C12—C7—C8—C9	−1.1 (3)	C7—N1—S1—C1	−61.80 (15)
N1—C7—C8—C9	−179.75 (16)	C6—C1—S1—O1	26.4 (2)
C7—C8—C9—C10	2.5 (3)	C2—C1—S1—O1	−156.35 (18)
C7—C8—C9—C14	−177.67 (19)	C6—C1—S1—O2	157.99 (18)
C8—C9—C10—C11	−2.3 (3)	C2—C1—S1—O2	−24.8 (2)
C14—C9—C10—C11	177.9 (2)	C6—C1—S1—N1	−87.25 (19)
C8—C9—C10—C15	178.14 (19)	C2—C1—S1—N1	89.97 (19)

### Hydrogen-bond geometry (Å, °)

**Table:**

*D*—H···*A*	*D*—H	H···*A*	*D*···*A*	*D*—H···*A*
N1—H1N···O2^i^	0.86	2.42	2.963 (2)	122

Symmetry codes: (i) *x*, −*y*+1/2, *z*−1/2.

## References

[bb1] Gelbrich, T., Hursthouse, M. B. & Threlfall, T. L. (2007). *Acta Cryst.* B**63**, 621–632. 10.1107/S010876810701395X 17641433

[bb2] Gowda, B. T., Foro, S., Babitha, K. S. & Fuess, H. (2008*a*). *Acta Cryst.* E**64**, o1691. 10.1107/S1600536808024653 PMC2960643 21201680

[bb3] Gowda, B. T., Foro, S., Babitha, K. S. & Fuess, H. (2008*b*). *Acta Cryst.* E**64**, o2190. 10.1107/S1600536808034351 PMC2959669 21581048

[bb4] Gowda, B. T., Foro, S., Babitha, K. S. & Fuess, H. (2009). *Acta Cryst.* E**65**, o366. 10.1107/S1600536809002098 PMC2968343 21581964

[bb5] Oxford Diffraction (2004). *CrysAlis CCD* Oxford Diffraction Ltd, Köln, Germany.

[bb6] Oxford Diffraction (2007). *CrysAlis RED* Oxford Diffraction Ltd, Köln, Germany.

[bb7] Perlovich, G. L., Tkachev, V. V., Schaper, K.-J. & Raevsky, O. A. (2006). *Acta Cryst.* E**62**, o780–o782.

[bb8] Sheldrick, G. M. (2008). *Acta Cryst.* A**64**, 112–122. 10.1107/S0108767307043930 18156677

[bb9] Shetty, M. & Gowda, B. T. (2005). *Z. Naturforsch. Teil A*, **60**, 113–120.

[bb10] Spek, A. L. (2009). *Acta Cryst.* D**65**, 148–155. 10.1107/S090744490804362X PMC2631630 19171970

